# Comparison of the Pathogenicity of Nipah Virus Isolates from Bangladesh and Malaysia in the Syrian Hamster

**DOI:** 10.1371/journal.pntd.0002024

**Published:** 2013-01-17

**Authors:** Blair L. DeBuysscher, Emmie de Wit, Vincent J. Munster, Dana Scott, Heinz Feldmann, Joseph Prescott

**Affiliations:** 1 Laboratory of Virology, Division of Intramural Research, National Institute of Allergy and Infectious Diseases, National Institutes of Health, Rocky Mountain Laboratories, Hamilton, Montana, United States of America; 2 Division of Biological Sciences, University of Montana, Missoula, Montana, United States of America; 3 Rocky Mountain Veterinary Branch, Division of Intramural Research, National Institute of Allergy and Infectious Diseases, National Institutes of Health, Rocky Mountain Laboratories, Hamilton, Montana, United States of America; University of Texas Medical Branch, United States of America

## Abstract

Nipah virus is a zoonotic pathogen that causes severe disease in humans. The mechanisms of pathogenesis are not well described. The first Nipah virus outbreak occurred in Malaysia, where human disease had a strong neurological component. Subsequent outbreaks have occurred in Bangladesh and India and transmission and disease processes in these outbreaks appear to be different from those of the Malaysian outbreak. Until this point, virtually all Nipah virus studies *in vitro* and *in vivo*, including vaccine and pathogenesis studies, have utilized a virus isolate from the original Malaysian outbreak (NiV-M). To investigate potential differences between NiV-M and a Nipah virus isolate from Bangladesh (NiV-B), we compared NiV-M and NiV-B infection *in vitro* and *in vivo*. In hamster kidney cells, NiV-M-infection resulted in extensive syncytia formation and cytopathic effects, whereas NiV-B-infection resulted in little to no morphological changes. *In vivo*, NiV-M-infected Syrian hamsters had accelerated virus replication, pathology and death when compared to NiV-B-infected animals. NiV-M infection also resulted in the activation of host immune response genes at an earlier time point. Pathogenicity was not only a result of direct effects of virus replication, but likely also had an immunopathogenic component. The differences observed between NiV-M and NiV-B pathogeneis in hamsters may relate to differences observed in human cases. Characterization of the hamster model for NiV-B infection allows for further research of the strain of Nipah virus responsible for the more recent outbreaks in humans. This model can be used to study NiV-B pathogenesis, transmission, and countermeasures that could be used to control outbreaks.

## Author Summary

Nipah virus causes severe disease in humans and outbreaks have occurred in two geographic regions, Malaysia and Bangladesh, and viruses have been isolated during outbreaks from both of these regions (NiV-M and NiV-B, respectively). The original outbreak of Nipah virus occurred in Malaysia and caused severe encephalitis in humans. All subsequent outbreaks of Nipah virus have occurred in Bangladesh or India and disease has been characterized as having a strong respiratory component. Nipah virus is a public health concern that can cause up to 100% lethality in humans and there is no approved treatment or vaccine. Current research should focus on understanding disease progression and pathogenicity. We compared NiV-M and NiV-B infection and disease progression using the Syrian hamster model. We found that NiV-M is more destructive in cultured hamster cells and has faster onset of cytopathogenicity compared to NiV-B. This is also true in hamsters, where although both viruses are pathogenic and cause a similar disease, pathology caused by NiV-M infection is accelerated. These data show that there is a difference in disease progression between the two strains of Nipah virus and will allow for a more detailed understanding of the events leading to disease caused by these viruses.

## Introduction

Nipah virus is a member of the family *Paramyxoviridae*, genus *Henipavirus*, and was discovered in 1998–99 to be the etiological agent responsible for an outbreak of severe respiratory disease in pigs [1] and encephalitis in humans in Malaysia [2]. All subsequent outbreaks of Nipah virus have occurred in Bangladesh or India, beginning in 2001, and have occurred on an almost annual basis [3]. Genetic data demonstrate that the isolates from Malaysia (NiV-M) and Bangladesh (NiV-B) represent two distinct Nipah virus strains [3], [4]. Nipah virus outbreaks have case fatality rates of up to 100% and there are no approved vaccines or treatments and these viruses have been categorized as a biosafety level 4 (BSL4) agents. Nipah virus differs from other paramyxoviruses in its ability to infect a wide range of mammals including bats [5], dogs [1], [6], horses [7], pigs [1], and cats [1], [8]. Wildlife surveillance at the time of the first outbreaks, along with several subsequent studies, has identified fruit bats of the family *Pteropodidae* as the natural reservoir of Nipah virus [5], [9]–[11].

During the first Nipah virus outbreak in Malaysia, NiV-M caused over 265 cases of encephalitis with 105 human deaths, resulting in a case fatality rate of 40% [1]. Common clinical manifestations of Nipah virus infection included fever, headache, respiratory disease, encephalitis and loss of consciousness [12], [13]. Fatal human cases of NiV-M infection were characterized by pathology involving the respiratory tract, central nervous system (CNS), heart, kidney and spleen [13]. NiV-M infection causes vasculitis characterized by destruction of the endothelium, syncytia formation, thrombosis and necrosis, with infiltration of inflammatory cells throughout affected organs. In the lungs of infected humans, pulmonary edema, alveolar hemorrhage and pneumonia were documented as well as occasional multinucleated giant cells found in alveolar space [13]. During this outbreak, the disease predominantly affected the nervous system with prominent signs of brain stem dysfunction. Magnetic resonance imaging of the brains of infected individuals showed focal lesions throughout the white matter [13]. In a study examining 94 Nipah virus-infected patients in Malaysia, only 6% showed abnormal chest radiographs, and of these, only one presented with a cough [2]. Also, cases of late onset or relapsing encephalitis were documented during the Malaysia outbreak [2]. During the Malaysian outbreak, pigs predominantly showed signs of respiratory disease and were determined to be an intermediate host [14], [15].

Epidemiologically, reports of infection with NiV-B differ from that of NiV-M infection in several aspects. Clinically, NiV-B infection resulted in a higher percentage of respiratory disease and a higher case fatality rate, reaching up to 100%, compared to NiV-M infection [12]. This disparity could reflect the differences in availability of health care and in reporting [16]. Disparities, however, could also be caused by intrinsic differences in the pathogenicity of NiV-M and NiV-B. NiV-B is transmitted from bats to humans by multiple routes including the ingestion of contaminated date palm sap [17], and can subsequently be transmitted nosocomially [18], or by human-to-human transmission [19]–[22]. Common clinical signs and symptoms of NiV-B infection included fever, altered mental status, headaches, cough, and difficulty breathing [16], [23]. During the Bangladeshi outbreaks, acute respiratory distress was noted in many patients [16], [24]. Febrile neurologic illnesses were also documented in some outbreaks of NiV-B, with lesions found in the gray and white matter of the brain [23], [25], [26]. In one study looking at 92 patients, 69% had difficulty breathing and 62% had a cough [16]. Limited studies have been conducted to describe the pathology in NiV-B infected patients.

In contrast to most other paramyxoviruses, Nipah virus has a broad species tropism and there are few suitable animal models that recapitulate human disease. Experimentally cats, guinea pigs, ferrets, pigs, non-human primates, and Syrian hamsters have been shown to support NiV-M viral replication resulting in clinical signs of infection [27]–[29]. The Syrian hamster is the only small rodent model that closely mimics multiple aspects of human disease [8], [27], [30], [31]. When infected intraperitoneally (i.p.) or intranasally (i.n.) with NiV-M, hamsters develop respiratory disease and/or encephalitis. The pathological changes that occur in the hamster are similar to those described in humans. The presence of vasculitis, necrosis, and inflammation is seen in both the human and hamsters. Viral antigen and disease pathology is observed in lung, kidney and heart tissue [31], [32]. Similar to human infections that lead to encephalitis, hamsters show antigen positive neurons, necrosis, and vasculitis in the CNS [31]. These similarities in infection between humans and hamsters make the Syrian hamster a suitable model for the study of Nipah virus pathogenesis.

This study was designed to compare NiV-B and NiV-M infections in a hamster-derived cell line, followed by a comparison of the pathogenesis and immune responses to infection by both virus strains in the Syrian hamster. Our results demonstrated that hamster cells are permissive for infection by both virus strains, with NiV-M causing increased syncytia formation and cytopathic effect (CPE) compared to NiV-B. *In vivo*, NiV-B infection resulted in a delayed disease progression compared to NiV-M infection. Overall NiV-M is more cytopathic *in vitro* and causes an accelerated disease *in vivo*, compared to NiV-B.

## Materials and Methods

### Ethical statement

All work with Nipah virus, potentially infectious materials, and infected hamsters was completed in the BSL4 facility at the Rocky Mountain Laboratories, Division of Intramural Research, National Institute of Allergy and Infectious Diseases, National Institutes of Health. All standard operating procedures applied were approved by the Institutional Biosafety Committee (IBC). All animal experiments were approved by the Institutional Animal Care and Use Committee of the Rocky Mountain Laboratories and performed following the guidelines of the Association for Assessment and Accreditation of Laboratory Animal Care, International (AAALAC) by certified staff in an AAALAC-approved facility.

### Virus propagation

NiV-B and NiV-M were provided by the Special Pathogens Branch of the Center for Disease Control and Prevention, Atlanta, GA, USA. NiV-M was isolated from a human case (cerebrum) in 1999 and passaged on Vero E6 cells a total of four times before used in experiments [33]. NiV-B was isolated from a throat swab of a lethal human infection from Bangladesh in 2004 and passaged in Vero E6 cells a total of three times [4]. Viruses were propagated on Vero E6 cells in Dulbecco's minimal essential medium (DMEM) (Sigma) supplemented with 10% fetal calf serum, 2 mM l-glutamine, 50 IU/mL penicillin and 50 µg/mL streptomycin (Life Technologies). Supernatants were collected and clarified by low-speed centrifugation and stored in liquid nitrogen.

### Virus titration

For plaque assays, Vero C1008 (European Collection of Cell Cultures) were grown to confluency in 6-well plates. Media was replaced with 250 µL of serial 10-fold dilutions of virus in DMEM and incubated for 1 hr at 37°C, rocking every 15 min. The virus inoculum was replaced with 2 mL of a 1∶1 mixture of 2× minimal essential medium (MEM) and 1.6% low-melt agarose (Life Technologies). The cells were then incubated for 3 d at 37°C, 5% CO_2_ before staining with 2 mL of a 0.25% crystal violet solution in 10% formalin for 3 hr at room temperature. The stain and overlay were then removed from the wells and the plaques were enumerated.

To determine the 50% tissue culture infectious dose (TCID_50_), monolayers of Vero C1008 cells were grown in 96-well plates and 100 µL of serial 10-fold diluted samples in MEM containing 2% FBS, were added to the wells. Cells were then incubated for 5 d at 37°C, 5% CO_2_ and then scored for CPE.

### Cell lines and *in vitro* infections

Baby hamster kidney cells (BHK-21) from the American Type Culture Collection were propagated in MEM (Sigma) supplemented with 10% fetal calf serum, 2 mM l-glutamine, 50 IU/mL penicillin and 50 µg/mL streptomycin (Life Technologies). Nipah virus infections were performed in 48-well plates when cells reached 95–100% confluency. Infections were performed by replacing medium with 250 µL of diluted virus (multiplicity of infection (MOI) of 0.1 and 0.01) in MEM, 2% FBS. After 1 hr, the inoculum was replaced with MEM supplemented with 2% FBS. Supernatants were collected at 1 hr, and 1, 2, and 3 days post infection (dpi) for virus titration. Cells were stained using the Kwik Diff Kit (Thermo scientific) to visualize syncytia according to the instructions of the manufacturer. Cells were monitored for CPE with a light microscope and images were captured using a Nikon DS-Fi1 camera.

### Inoculation of hamsters and sample collection

Groups of 5 to 6 week old female Syrian hamsters (Harlan) were inoculated with the indicated doses of NiV-M or NiV-B diluted in sterile DMEM and administered via the i.p. route in a total volume of 500 µL. Control animals received the equivalent volume of sterile DMEM by the same route. Two groups were inoculated i.n. with 105 TCID50 per hamster of either NiV-M or NiV-B diluted in sterile DMEM. Fifty microliters of virus preparation was delivered to each nare using a pipette. Hamsters were weighed and scored daily for clinical signs for two weeks. When signs of disease no longer existed, animals were monitored but no longer weighed. The health of animals was assessed and scored according to the following criteria: 0 = no signs of disease; 1 = ruffled fur; 2 = ruffled fur & weight loss <5%; 3 = ruffled fur, hunched posture & weight loss >5%; 4 = ruffled fur, hunched posture & weight loss >10%; 5 = ruffled fur, hunched posture, weight loss >15%, or encephalitic signs, or hemorrhagic signs, or paralytic signs or dyspnea; 6 = ruffled fur, hunched posture, weight loss >20%, or encephalitic signs, or hemorrhagic signs, or paralytic signs or dyspnea; 7 = death. Euthanasia occurred at a score of 5 and above. At the time of euthanasia, animals were bled (EDTA- and heparin-treated vacutainer tubes) via cardiac puncture. Necropsies were performed to collect lung, spleen, heart and brain tissue. Tissues were placed in lysis buffer RLT (Qiagen) for RNA extraction, or 10% formalin for histopathology and immunohistochemistry (IHC) analysis.

### RNA extraction and quantitative real-time RT-PCR (qRT-PCR)

Tissues (30 mg pieces) were homogenized in RLT buffer and removed from the BSL4 using approved standard operating procedures. Total RNA was extracted using RNeasy kit (Qiagen), according to the manufacturers' instructions. Whole blood was collected and inactivated in AVL buffer and removed from the BSL4 using approved standard operating procedures. Total RNA was extracted using QIAamp viral RNA kit (Qiagen), according to the manufacturers' instructions.

The RNA was quantified on a nanodrop 8000 spectrophotometer (Thermo Scientific). Real-time quantitative RT-PCR (qRT-PCR) was performed on a rotor-gene 6000 instrument (Corbett Life Science) using QuantiFast probe reagents (Qiagen) targeting the NiV-M or NiV-B nucleocapsid protein gene. Primers and probes used were: NiV-B sense (5′-GTTCAGGCCAGAGAAGCTAAATTT-3′), NiV-B antisense (5′-CCTCTTCGTCGACATCTTGATCA-3′), NiV-M sense (5′- GTTCAGGCTAGAGAGGCAAAATTT-3′), NiV-M antisense (5′- CCCCTTCATCGATATCTTGATCA-3″), NiV-B probe (5′-6FAM-CTGCAGGAGGTGTGCTCATCGGAGG-TAMRA-3′) and NiV-M probe (5′-6FAM-CTGCAGGAGGTGTGCTCATTGGAGG-TAMRA-3″). qRT-PCR components were used at the concentrations recommended by the manufacturer and 5 µL of RNA was added to each reaction and the following thermocycling parameters were used: 50°C for 10 min, 95°C for 5 min, and 40 cycles of 95°C for 5 s, 60°C for 10 s. Dilutions of RNA extracted from a known titer of each Nipah virus were run in triplicate to generate a standard curve from which sample TCID_50_ equivalents were extrapolated. Hamster immune gene expression was determined as previously described [34]. Briefly, RNA was extracted from tissues and qRT-PCR was performed as described above using gene-specific primers and probes under multiplex conditions. The fold-change in each gene was calculated by normalizing the change in C_T_ (ΔC_T_) to the C_T_ values for RPL18 (as an internal reference gene) for each sample and comparing this to the CT values of uninfected hamsters (2^−ΔΔCT^).

### Histopathology and immunohistochemistry

Tissues were fixed in 10% neutral buffered formalin for 7 d with one volume change, then transferred out of the BSL4 using approved standard operating procedures. Tissues were then placed in cassettes and processed with a Sakura VIP-5 Tissue Tek, on a 12 hr automated schedule, using a graded series of ethanol, xylene, and ParaPlast Extra. Embedded tissues were sectioned at 5 µm and dried overnight at 42°C prior to staining with hematoxylin and eosin (H&E).

Specific Nipah virus IHC was performed using an anti-Nipah virus N protein rabbit primary antibody at a 1∶5000 dilution (kindly provided by L. Wang, CSIRO Livestock Industries, Australian Animal Health Laboratory, Australia) [35]. The tissues were then processed using the Discovery XT automated processor (Ventana Medical Systems) with a DAPMap (Ventana Medical Systems) kit.

### Statistics

Statistical analyses were performed on the data form the TCID_50_ and qRT-PCR experiments using a 2-way ANOVA with a Bonferroni's post-test. To determine whether there were significant differences in the time to death between the viruses, we performed a log-rank test. The mean and SEM is represented and significance (* = p<0.05, ** = p<0.01 and *** = p<0.001) is reported where appropriate.

## Results

### NiV-M causes increased cytopathology in BHK-21 cells, compared to NiV-B

**Figure 1:**
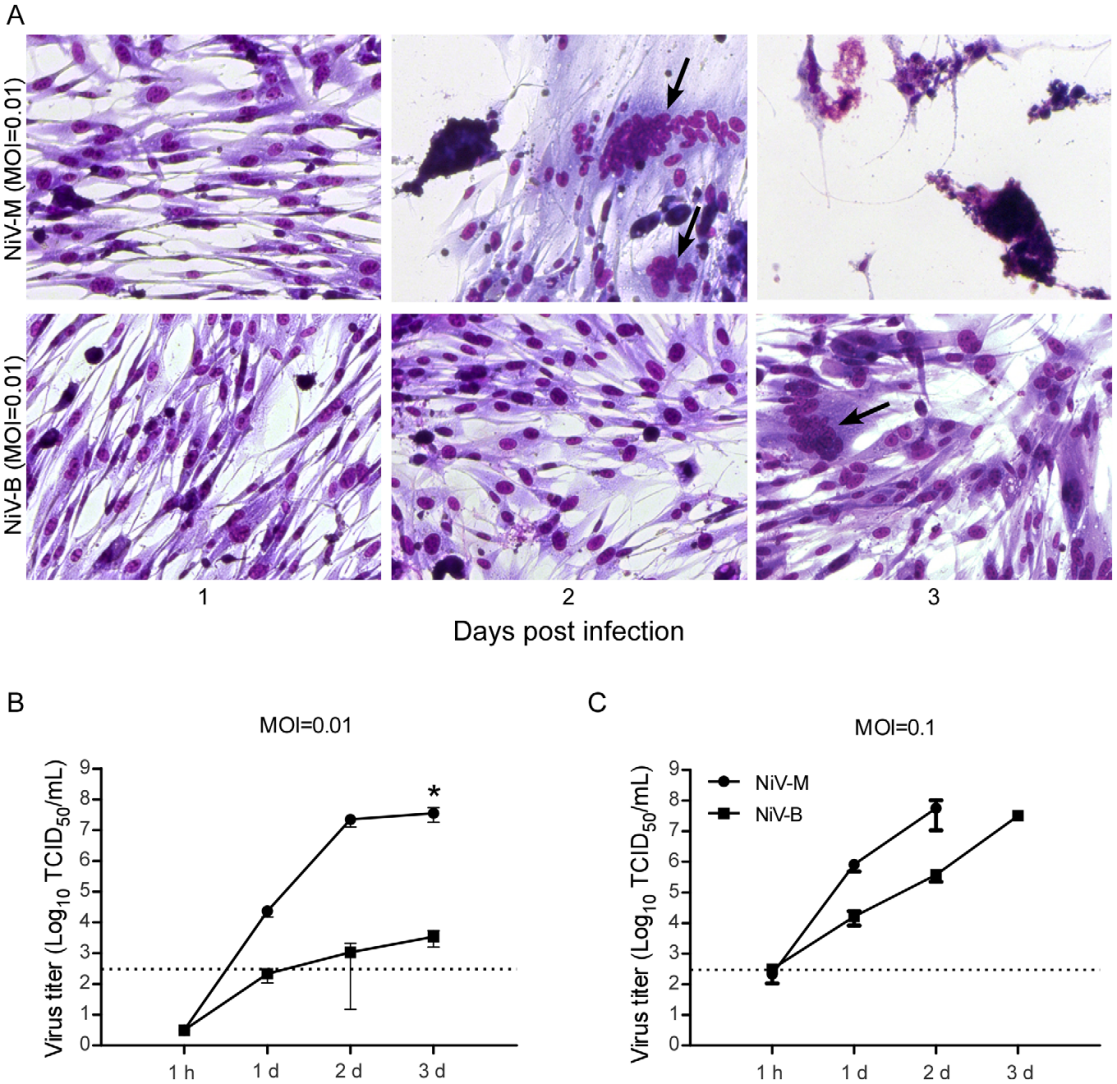
NiV-M replicates more efficiently and causes increased cytopathogenicity in hamster cells compared to NiV-B. To study the cytopathogenicity of these Nipah viruses, BHK-21 cells were infected with NiV-M or NiV-B at an MOI of 0.01 and stained using the Kwik Diff Kit at 1, 2, and 3 dpi (A). Arrows point to multinucleated giant cells. To examine the viral growth kinetics, BHK-21 cells were infected with Nipah virus at an MOI of 0.01 (B) or 0.1 (C). and supernatants were collected at the indicated time points. Supernatants of NiV-M at an MOI of 0.1 at 3 dpi were not collected due to extensive destruction of the cell monolayer. Virus was titrated on Vero C1008 cells and the results are expressed as the mean of three replicates and error bars indicate the SEM. The dotted line denotes limit of detection for the assay. A 2-way ANOVA with Bonferroni's post test was used to compare the viruses (* = p<0.05).

To determine the cellular responses and replication kinetics of the two Nipah virus strains in a hamster cell line, we infected BHK-21 cells with either NiV-M or NiV-B at MOIs of either 0.01 or 0.1. As early as 1 dpi, syncytia formation was apparent in all NiV-M-infected cultures. By 3 dpi, and at both MOIs, NiV-M-infected cells showed extensive CPE and nearly complete destruction of the cell monolayer (Figure 1A). NiV-B-infected cells showed little CPE at any of the time points sampled, regardless of the inoculation dose. At 3 dpi, NiV-B-infected cells began to form small syncytia. At both MOIs, NiV-M replicated sooner and reached higher virus titers in the supernatant at earlier time points compared to NiV-B (Figure 1B and C). At the lower MOI, NiV-M reached a titer that was 4 logs higher at 3 dpi than NiV-B (Figure 1B), whereas end titers were similar for both Nipah virus strains at the higher MOI, with a faster progression for NiV-M (Figure 1C).

### Disease progression during NiV-B infection of hamsters is delayed compared to NiV-M infection

**Figure 2:**
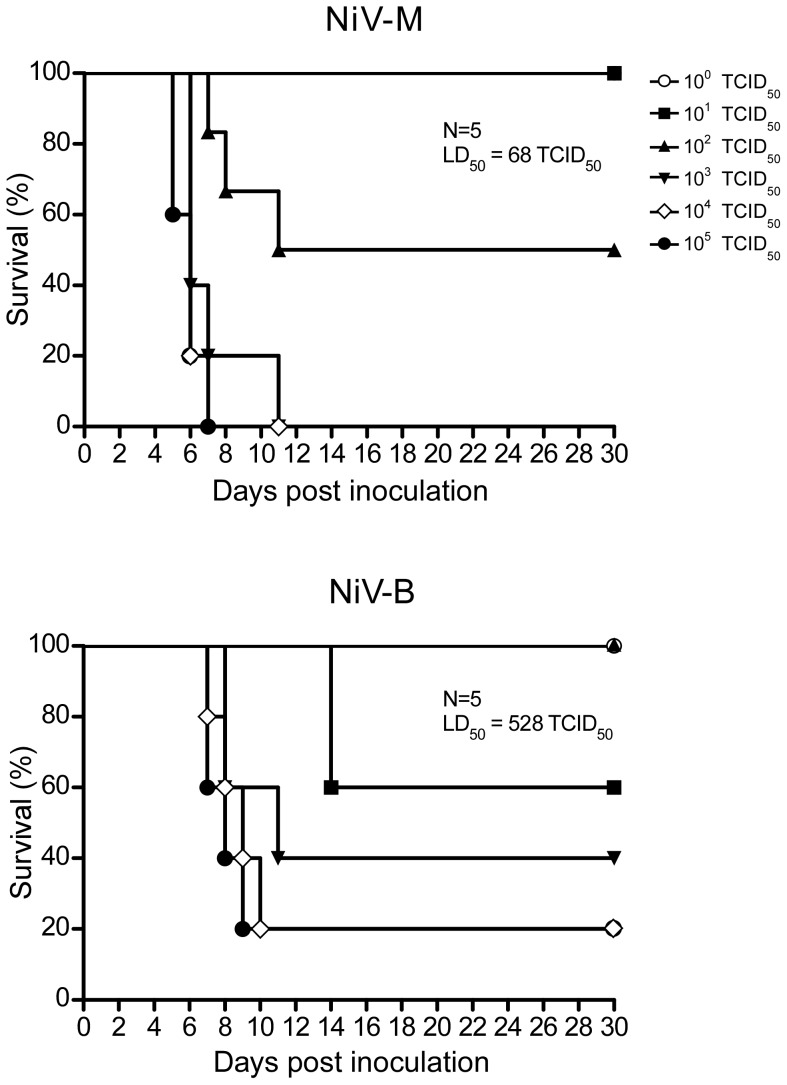
Hamsters inoculated with NiV-B show delayed disease progression compared to NiV-M-infected hamsters. Groups of 5 hamsters were inoculated i.p. with 10-fold serial dilutions of virus from 10^5^ to 1 TCID_50_. The hamsters were monitored for 30 dpi for survival.

To date, NiV-B infection has not been examined in an animal model. To assess the suitability of the hamster as a model for NiV-B infection, as well as to compare the two strains, hamsters were inoculated i.p. with 10-fold serial dilutions of Nipah virus from 10^5^ to 1 TCID_50_ (Figure 2). Animals were evaluated for clinical signs of disease on a daily bases according to a scoring system outlined in the Materials and Methods section. Only one hamster showed abnormal clinical signs on the day prior to euthanasia, which consisted of ruffled fur. All other hamsters did not display abnormal clinical signs until the day euthanasia was necessary. Hamsters challenged with either virus strain showed clinical signs of respiratory distress and/or neurologic dysfunction leading to a score that required euthanasia. Signs of respiratory disease included labored abdominal breathing and hunched posture; neurological dysfunction included imbalance, partial paralysis and seizures. Similar to previous studies with NiV-M, respiratory distress was observed only in animals infected at the highest doses (10^4^ and 10^5^ TCID_50_/animal) [32]. The majority of animals inoculated with the lower doses of Nipah virus (10^0^ through 10^3^ TCID_50_/animal) displayed neurologic dysfunction prior to euthanasia. One animal infected with NiV-M at the highest dose (10^5^ TCID_50_) and two animals infected with NiV-B (one inoculated with 10^4^ and one with 10^5^) presented with both respiratory and neurologic dysfunction, while the rest of animals had either respiratory or neurological signs of distress that required euthanasia. NiV-M-infected animals showing severe respiratory signs of disease were euthanized between 5–7 dpi, whereas animals displaying neurological disorders were euthanized between days 5–11. Disease progression in NiV-B-infected animals was generally slower, and animals displaying severe respiratory distress or neurological dysfunction were euthanized on 8–9 dpi or 8–14 dpi for NiV-M and NiV-B infection, respectively (Table 1). The slower disease progression in NiV-B-infected animals was reflected in the overall survival curves with 80% lethal disease outcome even at the highest dose of infection (Figure 2 and Table 1). The LD_50_ for NiV-M and NiV-B was 68 and 528 TCID_50_, respectively. At both 10^3^ and 10^5^ TCID_50_, there was a statistically significant difference in the time to death between the two virus strains, with death occurring approximately two days later for NiV-B infected animals at each dose (Table 1). To determine if the delay in survival is associated with the route of infection, we inoculated hamsters i.n. with 10^5^ TCID_50_ of either NiV-M or NiV-B. The mean time to death was delayed by two days in hamsters inoculated with NiV-B compared to NiV-M (Figure S1). Both routes of inoculation showed a two-day delay in NiV-B compared to NiV-M, although the mean time to death was later in both virus groups with the i.n. compared to i.p. route.

**Table 1:** Clinical signs and outcome of hamsters inoculated with NiV-M or NiV-B.

Inoculum	Dose (TCID_50_)	Survival (%)	Mean time to death (days)^a^
NiV-M	1×10^0^	100	N/A
	1×10^1^	100	N/A
	1×10^2^	40	8.7±1.7
	1×10^3^	0	7.2±1.9* b
	1×10^4^	0	7±2
	1×10^5^	0	5.8±0.7** c
NiV-B	1×10^0^	100	N/A
	1×10^1^	60	14±0
	1×10^2^	100	N/A
	1×10^3^	40	9±1.4* b
	1×10^4^	20	8.5±1.1
	1×10^5^	20	7.75±0.8** c

a Excluding survivors.

b Significant difference between NiV-M and NiV-B at dose 10^3^ TCID_50_,

* p<0.05: Log-rank test.

c Significant difference between NiV-M and NiV-B at dose 10^5^ TCID_50_,

** p<0.01: Log-rank test.

### Both Nipah viruses replicate in hamster lung, brain and spleen tissue

**Figure 3:**
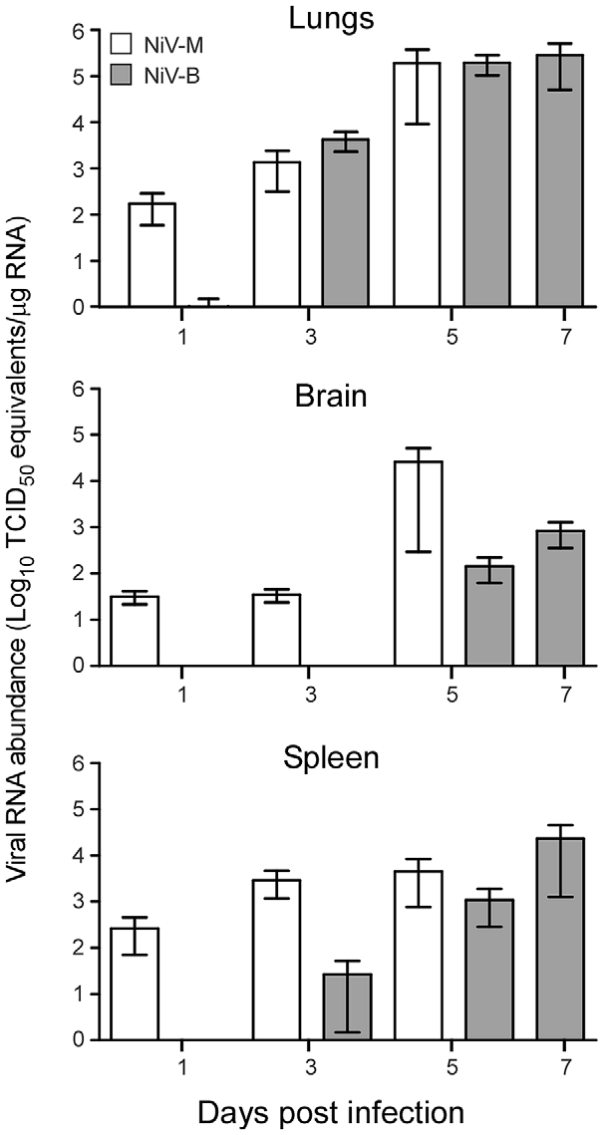
NiV-B replication is delayed in hamster organs compared to NiV-M replication. Hamsters were inoculated with 10^5^ TCID_50_ of Nipah virus and 9 animals/group were euthanized at 1, 3, 5, and 7 (for NiV-B only) dpi and tissues were collected. Total viral RNA was extracted and Nipah virus N-specific viral RNA was quantified by qRT-PCR. Gray bars represent NiV-B and white NiV-M. Bars represent the mean and error bars represent the SEM.

To compare the pathogenesis of NiV-M to NiV-B, groups of hamsters were inoculated with 10^5^ TCID_50_ of either Nipah virus and tissues were collected on 1, 3 and 5 dpi for both virus groups, and 7 dpi for NiV-B. Based on the time to death at this dose from our survival experiment, 7 dpi tissues were not collected for NiV-M-inoculated animals for this pathology experiment. Viral RNA was detected using Nipah virus N-specific qRT-PCR (Figure 3). In NiV-M-inoculated animals, replication was detected at an earlier time point than NiV-B replication. As early as 1 dpi, viral RNA was detected in lungs, brain and spleen tissue of some NiV-M-infected animals. NiV-B-infected animals had detectable levels of viral RNA at 1 dpi in lung tissue of some hamsters, and in the spleen by 3 dpi. Both strains showed an increase in viral RNA over time in the lungs, brain and spleen, with the highest overall titers in the lungs at the last time point sampled. We assessed viremia in hamsters inoculated with either virus by qRT-PCR. Levels of viral RNA were barely detectable and viral RNA was undetectable in some animals at each time point (data not shown).

### Host gene expression in lung, brain and spleen tissue of hamsters is differentially regulated during Nipah virus infection

**Figure 4:**
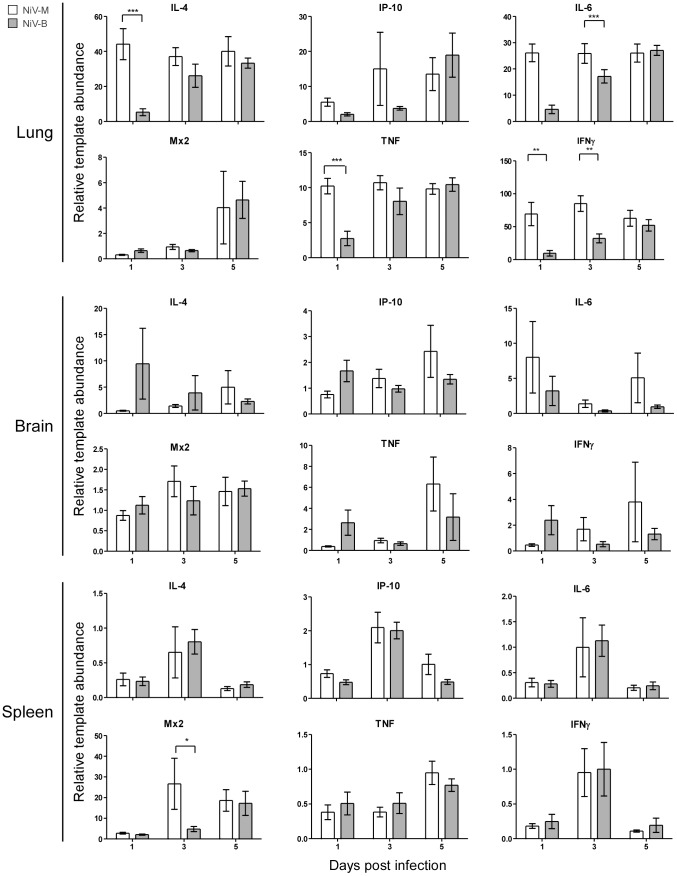
Host gene expression in lung, brain and spleen tissue of hamsters is differentially regulated during infection with Nipah viruses. Quantitative RT-PCR for IL-4, IP-10, IL-6, Mx2, TNF and IFNγ was performed on lung, brain and spleen tissues from groups of 9 hamsters inoculated with 10^5^ TCID_50_ of NiV-M (white bars) or NiV-B (gray bars) at the indicated time points. Data are shown as the fold-change of each gene over uninfected controls and normalized to an internal reference gene (RPL18). Error bars represent the SEM. A 2-way ANOVA with Bonferroni's post-test was used to determine statistical significance between viruses (* = p<0.05, ** = p<0.01 and *** = p<0.001).

To examine the kinetics of the host immune response to Nipah virus infection, and compare responses between NiV-M and NiV-B infections, the expression level of a subset of cytokine and chemokine mRNAs were examined by qRT-PCR in the lungs, brain and spleen (Figure 4). Throughout the infection, the largest overall response was seen in the lungs. At 1 dpi, a statistical difference in the upregulation of interleukin-4 (IL-4), interleukin-6 (IL-6), tumor necrosis factor (TNF), and interferon-γ (IFNγ) was observed between NiV-M and NiV-B infections, with higher expression of these genes in response to NiV-M. A similar result was measured at 3 dpi for IFNγ in the lungs (Figure 4). At 3 dpi, IL-4, IL-6 and TNF were upregulated similarly in response to both virus strains and remained upregulated throughout the course of infection. Upregulation of the gene for myxovirus resistance protein-2 (Mx2) in the lungs was detected only at the last time point for both virus strains. IFNγ-induced protein 10 (IP-10) mRNA increased starting at 1 dpi and remained upregulated in the lungs throughout the course of infection, peaking at 3 dpi in NiV-M-infected hamsters and 5 dpi in NiV-B-infected hamsters. IL-4, IL-6 and TNF were also upregulated in brain (Figure 4). There was a significant increase in Mx2 transcription in the spleens of NiV-M-infected hamsters at 3 dpi compared to NiV-B. In the brain, IL-4, IL-6 and TNF were slightly upregulated over control animals. IL-4, IL-6, TNF, and IFNγ mRNAs were downregulated in the spleen.

### Histopathological changes occurred earlier in NiV-M-infected hamsters compared to NiV-B-infected animals

**Figure 5:**
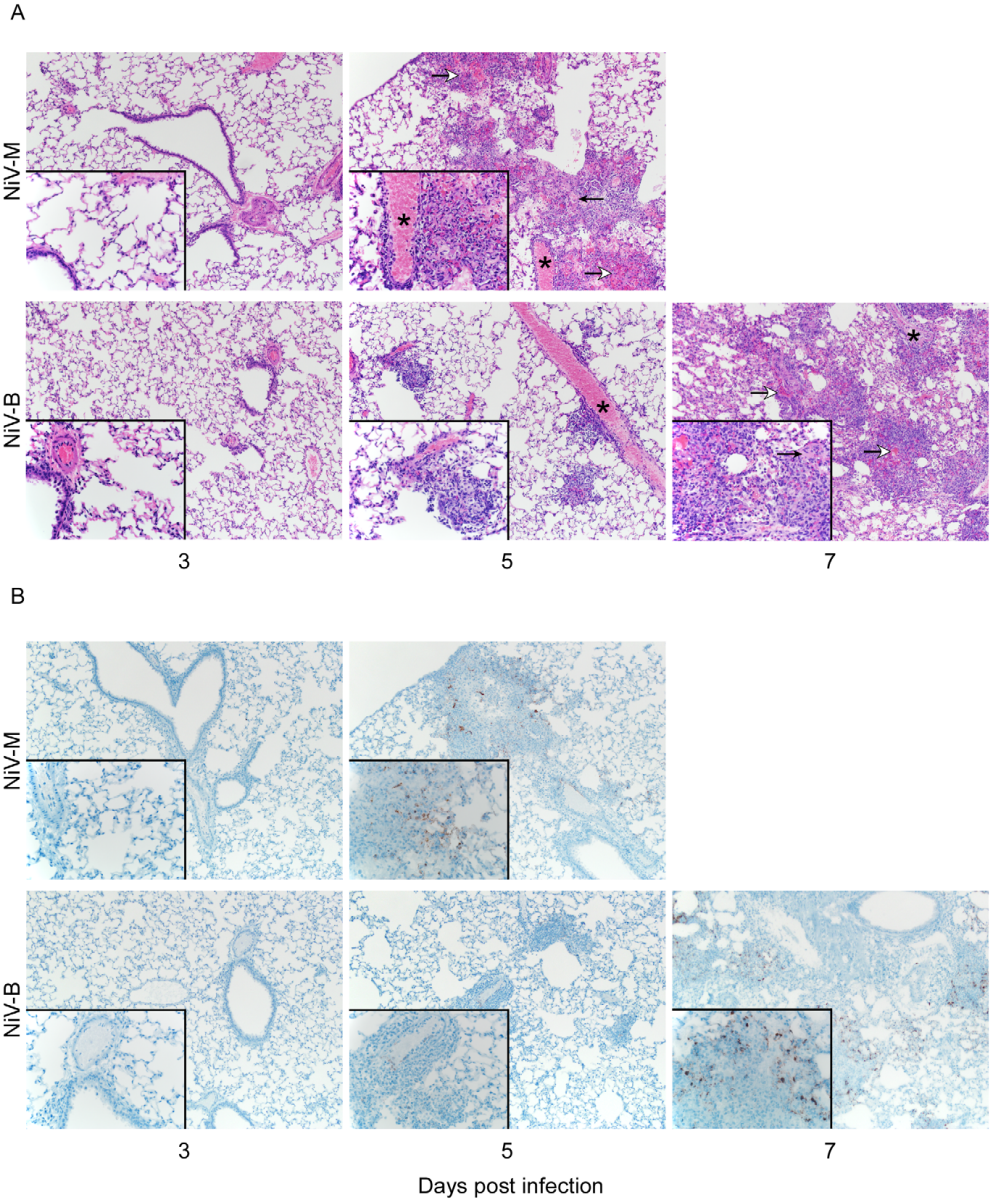
NiV-M infection results in accelerated pathology compared to NiV-B infection in hamsters. Nipah virus inoculated hamsters were euthanized at 3, 5 and 7 dpi (for NiV-B only) and lung sections were stained with H&E (A) and for Nipah virus nucleocapsid protein (IHC) (B) Images were taken at a magnification of 100× and 400× (insets). Asterisks denote arteries with vasculitis as demonstrated by recruitment of inflammatory cells with effacement of the tunica intima and tunica media. Open arrows denote areas of acute hemorrhage and closed arrows indicate fibrin deposits.

To compare the pathology between the two strains, hamsters were inoculated with 10^5^ TCID_50_ of NiV-B or NiV-M and tissues were examined histologically. Pathology was observed for both infections and was composed of a mild to moderate multifocal, subacute, bronchointerstitial pneumonia with vasculitis on 5 dpi for NiV-M and NiV-B infection (Figure 5). By 7 dpi in NiV-B-infected animals, the pneumonia progressed to marked, multifocal to coalescing, subacute bronchointerstitial pneumonia with vasculitis, necrosis, edema, and fibrin deposits. The pneumonia in both groups, on day 5 dpi for NiV-M infection and 7 dpi for NiV-B infection, was characterized by effacement of terminal bronchioles and adjacent alveoli by small to moderate numbers of macrophages, neutrophils, lymphocytes and plasma cells. Multifocal vasculitis was observed with disruption of the arterial tunica media by small numbers of neutrophils and lymphocytes. Syncytial endothelial cells were found in affected small to medium caliber vessels. Hamsters from the final time points had moderate to marked lesions in the lungs and demonstrated a loss of pulmonary architecture with replacement by cellular and karyorrhectic debris with small to moderate amounts of hemorrhage, fibrin deposits and edema. IHC revealed viral antigen in alveolar capillary endothelium, small and medium caliber arteriolar endothelium, and in mononuclear inflammatory cells starting at 3 dpi for NiV-M infection and 5 dpi for NiV-B infection (Figure 5B). The presence of viral antigen was strongly associated with areas of inflammation. No pathological changes were observed in the CNS of hamsters infected at the high dose used in the pathology study.

## Discussion

Nipah virus is a zoonotic pathogen that causes encephalitis and pulmonary disease with a high case fatality rate and is classified as a category C pathogen by the NIAID's pathogen priority list [12]. Two strains of Nipah virus, NiV-M and NiV-B, have been isolated from geographically and temporally separated outbreaks [4]. Human outbreaks caused by these strains differ in disease progression and epidemiologically [23]. The Syrian hamster has been established as a disease model for NiV-M infection [31], [32], but NiV-B infection studies have not been reported for any animal model. The goal of this study was to compare the replication, pathogenesis, and immune response to infection with NiV-M and NiV-B using *in vitro* and *in vivo* methods. BHK-21 cells infected with NiV-M showed more severe damage and supported higher virus replication compared to NiV-B-infected cells. Hamsters infected with NiV-B had a delay in disease progression and increased survival rates compared to NiV-M infected animals.

*In vitro*, BHK-21 cells were permissive for infection by both NiV-M and NiV-B. NiV-M replicated to higher titers in the supernatant at an earlier time point, and infection resulted in widespread syncytia formation causing extensive CPE. Widespread CPE was not observed in NiV-B-infected BHK-21 cells, although a few syncytia were present at later time points. The differences observed in virus replication and syncytia formation could be attributed to either higher viral replication causing more syncytia, or more syncytia formation resulting to higher virus load. Differences in replication efficiency including protein production, viral assembly and budding could explain the higher virus production and large number of syncytia observed in NiV-M infected cultures. Conversely, differences in fusion kinetics could account for disparate amounts of syncytia that then lead to variation in virus replication. The high amount of fusion and syncytia formation in cells could result in the infection of cells that were not initially infected at the low MOIs used in our experiments. This could lead to higher overall levels of virus production. Previous studies using paramyxoviruses have demonstrated that viral spread can occur by cell-cell fusion; the surface proteins of Nipah virus are present at the cell junctions and have been shown to initiate fusion and spread of virus [36]–[38]. Slower fusion kinetics could lead to less and slower formation of syncytia observed in NiV-B infected cells. The affinity of Nipah virus glycoprotein to its receptors, ephrin B2 and B3, as well as the ability of the glycoprotein to trigger the fusion protein could also affect fusion rates. Further experiments need to be completed to examine the relationship between replication and syncytia formation.

In our experiments, we chose i.p. as the route of inoculation due to the more uniform disease progression and outcomes described in previous Nipah virus studies [31]. It is likely that i.p. inoculation would more readily allow for the detection of subtle differences between strains that may not be detectable in a less uniform infection route, such as i.n. When infected with NiV-M or NiV-B, hamsters developed clinical signs of disease similar to human infection [2], [13], [31], [39]. The onset of disease and death in hamsters was rapid and occurred between 5–14 dpi, which corresponds to human cases, where symptoms start to develop between 7–10 dpi [15], [40], [41]. We observed earlier replication of NiV-M than NiV-B in all organ types sampled, although, once NiV-B RNA was detected, it reached similar values within two days. Earlier replication of NiV-M in tissues corresponded with earlier pathologic changes and accelerated disease and death compared to NiV-B infection. In humans, CNS pathology is documented, but in our comparative pathology experiment, we did not observe pathology in the CNS. This is likely attributed to the high dose of inoculum for the pathology experiment (10^5^ TCID_50_) and route of inoculation (i.p.). However, we did observe pathology in the lungs consisting of multifocal subacute bronchointerstitial pneumonia with vasculitis. The pneumonia was characterized by inflammation in the terminal bronchioles and alveoli spaces, necrosis, hemorrhage, fibrin deposits, edema and syncytia in endothelial cells. In human cases, fibrinoid necrosis, vasculitis, pulmonary edema, alveoli hemorrhaging, and syncytia were documented [13], [15], [40]. It is probable that hamsters inoculated with this high dose (10^5^ TCID_50_) succumbed to infection due to inflammation, edema, and widespread vasculitis in the lungs that caused interstitial pneumonia. Even with low levels of viral antigen, pathology was severe enough to cause a fatal outcome.

The typical dose that humans are infected with, as well as the route of infection is not known. In hamsters, both virus strains caused respiratory distress and/or neurological dysfunction in a dose-dependent manner. Based on previous data in hamsters, it is likely that dose and route of infection might play a role in Nipah virus outcome in humans [32]. Disease progression could be altered by the transmission route, which could include fomite [18], [42], oral ingestion [17], [43], and respiratory droplets [42], [44], [45]. In this study, inoculation of hamsters with NiV-B resulted in a delay in disease progression and the LD_50_ was approximately a log higher compared to NiV-M. However these data are contrary to what has been reported in humans, where NiV-B results in higher case fatality rates compared to NiV-M. Since we did not observe a difference in disease that would explain differences in the epidemiological data for the two Nipah virus strains, factors other than the intrinsic pathogenicity likely contribute to the disparities in the documented epidemiological data. The suboptimal health care, lack of supportive care and inconsistencies in reporting could account for higher documented case fatality rates and differences in disease manifestations during NiV-B outbreaks [16].

Cytokine and chemokine mRNAs were quantitated in the hamsters over the course of infection and several immune genes were upregulated in the lung, brain, and spleen, although there was a slight downregulation of some genes in the spleen. NiV-M induced an earlier and more robust immune response compared to NiV-B, which eventually reached similar levels to hamsters infected with NiV-M. Early TNF activation during NiV-M infection may contribute to recruitment of inflammatory cells, as observed in the lungs of infected hamsters by histopathology. The upregulation of IP-10 in the lungs coincided with lymphocyte recruitment, appearance of vascular damage, and necrosis in the lungs. IP-10 upregulation has been documented in other Nipah virus studies, specifically focusing on endothelial cells [46], [47]. Teruya-Feldstein et al reported that high levels of IP-10 are found in necrotic tissue and in areas of vascular damage associated with Epstein-Barr virus-positive lymphoproliferative processes in mice [48]. They demonstrated a correlation between IP-10 regulation, tissue necrosis, and vascular damage during viral infection. Similarly, IP-10 is upregulated in the airways of patients with pulmonary diseases such as tuberculosis and plays a role in recruitment of activated T cells [49]. IL-6 gene expression was increased earlier in the lung in NiV-M compared to NiV-B infected hamsters. IL-6 activates T cells [50] and the recruitment of T cells likely contributed to the widespread vasculitis associated with Nipah virus infection and disease. Recruitment of lymphocytes could also be a way for Nipah virus to disseminate throughout the host, as it has recently been published that lymphocytes and monocytes can carry virus without becoming infected and release virus at distant sites from the original infection [51], [52]. In the lungs, IL-4 was also upregulated, following similar kinetics than IL-6. IL-4 promotes differentiation of B cells, and is upregulated is indicative of the activation of a Th2 response [53]. However, during disease, specific antibody production would not occur fast enough, since animals succumb to infection before significant antibody production can likely occur. Due to the use of the hamster as a model, we are limited in the amount of reagents available for a detailed examination of the immune response and future work is needed to get a more complete picture of the immune response to Nipah virus infection. In general NiV-M infection caused earlier induction of immune genes which probably corresponds to the earlier pathology observed. It is possible that the strong early immune response in Nipah virus-infected animals might contribute to disease via an immunopathogenic mechanism.

In conclusion, there is a delay in NiV-B-induced disease progression compared to NiV-M, specifically in time to death, virus replication, pathology and immune responses. NiV-M is more cytopathic *in vitro* and more pathogenic *in vivo*. Viral antigen staining was low in tissues, although the pathologic changes were extensive and the inflammatory response was robust, suggesting disease progression may not only be a result of direct effects of the virus, but likely has an immunopathogenic component. The experimental data presented herein characterizes the hamster as a suitable small animal model for NiV-B infection, showing clinical signs, viral tropism, and pathologic changes similar to those observed in humans. These data are important to further the understanding of Nipah virus infection and pathogenesis. By applying the hamster model for NiV-B this allows for future studies in transmission, pathology and therapeutics, specifically focusing on the Nipah virus strain responsible for recent outbreaks.

## Supporting Information

Figure S1 **Hamsters inoculated intranasally with NiV-B show delayed disease progression compared to NiV-M-inoculated hamsters.** Groups of 5 hamsters were inoculated i.n. with 10^5^ TCID_50_. The hamsters were monitored for survival. A log- rank test was used to compare survival curves (* = p<0.05). NiV-B infected animals had a mean time to death of 11.6 days and NiV-M infected animals 9.4 days.
(TIF)
