# Supplementary figures and images for: Comparison of the Pathogenicity of Nipah Virus Isolates from Bangladesh and Malaysia in the Syrian Hamster
Source: PLoS Negl Trop Dis. 2013 Jan 17;7(1):e2024. doi: 10.1371/journal.pntd.0002024 (PMC3547834; doi:10.1371/journal.pntd.0002024)

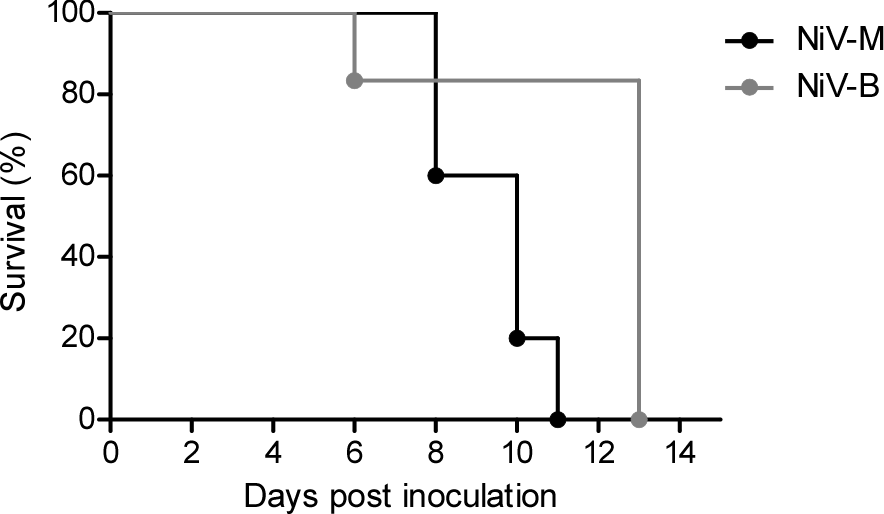

Supplement: Figure S1 — Hamsters inoculated intranasally with NiV-B show delayed disease progression compared to NiV-M-inoculated hamsters. Groups of 5 hamsters were inoculated i.n. with 105 TCID50. The hamsters were monitored for survival. A log- rank test was used to compare survival curves (* = p<0.05). NiV-B infected animals had a mean time to death of 11.6 days and NiV-M infected animals 9.4 days. (TIF) [file pntd.0002024.s001.tif]
